# Effects of the dose-volume relationship on and risk factors for maxillary osteoradionecrosis after carbon ion radiotherapy

**DOI:** 10.1186/1748-717X-9-92

**Published:** 2014-04-03

**Authors:** Go Sasahara, Masashi Koto, Hiroaki Ikawa, Azusa Hasegawa, Ryo Takagi, Yoshitaka Okamoto, Tadashi Kamada

**Affiliations:** 1 Department of Otorhinolaryngology, Head and Neck Surgery, Chiba University Graduate School of Medicine, Chiba, Japan; 2 Research Center for Charged Particle Therapy Hospital, National Institute of Radiological Sciences, Chiba, Japan

**Keywords:** Carbon ion radiotherapy, Osteoradionecrosis, Head and neck tumor, Dose-volume histogram

## Abstract

**Background:**

Osteoradionecrosis (ORN) is a critical complication after carbon ion (C-ion) or photon radiotherapy (RT) for head and neck tumors. However, the risk factors for ORN after C-ion RT remain unclear. Therefore, the present study aimed to investigate the effects of the dose-volume relationship on and risk factors for ORN development after C-ion RT. We, however, focused on the maxillary bone because most tumors treated with C-ion RT were primarily located in the sinonasal cavity.

**Methods:**

The patients enrolled in this study received more than 10% of the prescribed total dose of 57.6 Gy equivalent (GyE) in 16 fractions to their maxilla. All patients were followed up for more than 2 years after C-ion RT. Those with tumor invasion to the maxilla before C-ion RT or local recurrence after the treatment were excluded from the study to accurately evaluate the effects of irradiation on the bone. Sixty-three patients were finally selected. The severity of ORN was assessed according to the Common Terminology Criteria for Adverse Events version 4.0. The correlation between clinical and dosimetric parameters and ORN incidence was retrospectively analyzed.

**Results:**

The median follow-up period was 79 months. Of the 63 enrolled patients, 26 developed ORN of grade ≥1. Multivariate analysis revealed that the maxilla volume receiving more than 50 GyE (V50) and the presence of teeth within the planning target volume were significant risk factors for ORN. Dose-volume histogram analysis revealed that V10 to V50 parameters were significantly higher in patients with ORN than in those without ORN.

**Conclusions:**

V50 and the presence of teeth within the planning target volume were independent risk factors for the development of ORN after C-ion RT using a 16-fraction protocol.

## Background

Carbon ion radiotherapy (C-ion RT) offers a potential cure for radio-resistant tumors owing to its increased biologic potential and improved dose localization properties. In a recent report, we demonstrated that C-ion RT was a promising treatment option for various inoperable and radio-resistant tumors [1].

In addition, we have published the results of a phase II clinical trial of C-ion RT in patients with head and neck tumors [2]. In this trial, C-ion RT demonstrated excellent disease control in radio-resistant tumors such as mucosal malignant melanoma, adenoid cystic carcinoma, and adenocarcinoma. However, the development of osteoradionecrosis (ORN) remained one of the serious complications after C-ion RT.

ORN is a well-documented complication of photon RT for head and neck tumors. The degree of ORN varies from limited, asymptomatic bone exposures that may remain stable for a prolonged period of time or heal with conservative management, to severe necroses necessitating surgical intervention and reconstruction [3]. Thus, ORN is a serious clinical problem. Several clinical risk factors for ORN such as smoking, drinking, oral hygiene, and tumor characteristics have been implicated in the literature [3-5]. The radiation dose or dose-volume histogram (DVH) parameter has been also known to be a major contributor to ORN [4,6-9]. However, there remains a lack of data on ORN after C-ion RT.

The present study aimed to investigate the effects of the dose-volume relationship on and risk factors for ORN development after C-ion RT. We, however, focused on the maxillary bone in this investigation because most tumors treated with C-ion RT were located in the sinonasal cavity.

## Methods

### Patients and tumor characteristics

From April 1997 to February 2006, a total of 236 patients with head and neck tumors were treated with C-ion RT in a phase II clinical trial at our institution [2]. Of these, patients who received more than 10% of a prescribed total dose of 57.6 gray equivalent (GyE) in 16 fractions to the maxilla and were followed up for more than 2 years after C-ion RT were selected. Those with tumor invasion to the maxilla before C-ion RT or local recurrence after treatment were excluded from the study to accurately evaluate the effects of irradiation on the maxilla. Based on results from a previous clinical trial [10], a total dose of 57.6 GyE or 64.0 GyE in 16 fractions was prescribed for head and neck tumors. However, only patients treated with 57.6 GyE were included in the present study as a homogeneous population for analysis. Sixty-three patients were finally included in our study cohort.

Before C-ion RT, all patients provided written informed consent acknowledging the possibility of ORN as a result of the treatment. The study was approved by the institutional ethics committee (National Institute of Radiological Sciences).

The median age of the 63 enrolled patients (32 men and 31 women) was 59 years (range, 16–80 years). Of these, 22 smoked and 31 consumed alcohol. The presence of teeth within the planning target volume (PTV) was documented in 29 patients. Histological classification of all patients’ tumors included 24 patients of malignant melanoma, 24 adenoid cystic carcinomas, 9 adenocarcinomas, 2 squamous cell carcinomas, 2 mucoepidermoid carcinomas, and 1 each of epithelial/myoepithelial carcinoma and undifferentiated carcinoma. Eighteen of the studied tumors were located in the paranasal sinus, 17 in the nasal cavity, 12 on the parotid gland, 6 in the oral cavity, and 10 at other sites. All patients underwent dental examination and management before C-ion RT. If dental extraction was necessary, C-ion RT was performed at least 10 days after such procedure. Of the 23 patients who received adjuvant or neo-adjuvant chemotherapy, 18 had malignant mucosal melanoma and received combinatorial chemotherapy with dacarbazine, nimustine, and vincristine.

### C-ion RT

For C-ion RT, each patient was positioned in customized cradles (Moldcare; Alcare, Tokyo, Japan) and immobilized with a low-temperature thermoplastic device (Shellfitter; Kuraray, Osaka, Japan). A set of computed tomography (CT) images at 2.5-mm slide thickness was obtained for treatment planning using the immobilization devices. Three-dimensional treatment planning was performed with HIPLAN software [11].

The clinical target volume had a minimum margin of 5 mm added to the gross tumor volume. Furthermore, a margin of 3–5 mm was added as an internal and setup margin around the clinical target volume to create a final PTV. In principle, more than 3 portals were used to improve dose distribution.

During each treatment session, the patient’s position was verified with a computer-aided online positioning system. The patient was positioned on the treatment couch using immobilization devices, and then digital orthogonal radiographic images were taken and transferred to the positioning computer. The positioning images were compared with the reference images digitally reconstructed from CT scans. If the difference in positioning was more than 2 mm, the treatment couch was repositioned until appropriate.

The carbon beam dose was expressed in terms of photon equivalent dose, defined as the physical dose multiplied by the relative biological effectiveness (RBE) of carbon ions. The clinical RBE of the carbon beam at our institute was determined according to the RBE for acute skin reaction, which was 3.0 at the distal part of the spread-out Bragg peak [12]. C-ion RT was delivered in 16 fractions over a 4-week period with 4 treatment days per week. The prescribed total dose was 57.6 GyE in all patients. C-ion RT was performed without concurrent chemotherapy in all patients.

### Follow-up and ORN evaluation

**Figure 1:**
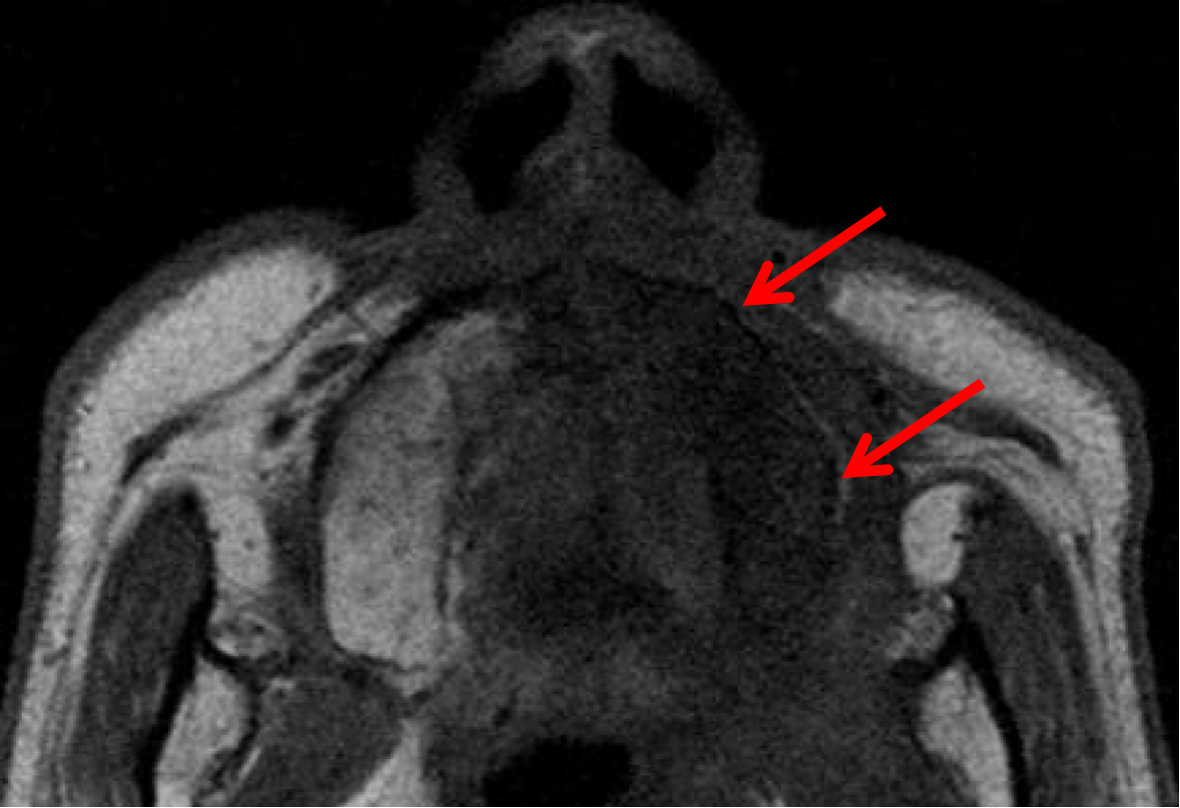
**Representative magnetic resonance image (MRI) of the maxillary osteoradionecrosis.** T1-weighted axial MRI showed low signal appearance in the left maxilla (red arrows).

After C-ion RT, patients were followed up every 2 to 3 months during the first 2 years and every 3 to 6 months thereafter. Magnetic resonance imaging (MRI) examination of the head and neck region was performed at 4–6-month intervals. A diagnosis of maxillary ORN was indicated on the basis of clinical symptoms, macroscopic observation, and MRI findings demonstrating an area of low intensity on T1-weighted images and/or that of high-intensity on T2-weighted images present within the irradiation field after C-ion RT (Figure 1). Enhancement on post-contrast images and diffusion-weighted images were used to exclude the possibility of local recurrence. The severity of ORN was assessed according to the Common Terminology Criteria for Adverse Events version 4.0 as follows: grade 1, asymptomatic with clinical or diagnostic observations only and intervention not indicated; grade 2, symptomatic, limiting instrumental activities of daily living, and medical intervention indicated; grade 3, severe symptoms, limiting self-care activities of daily living, and selective surgical intervention indicated; grade 4, life-threatening consequences with urgent intervention indicated. The highest grade was used for analysis.

### Dose-volume histogram analysis

**Figure 2:**
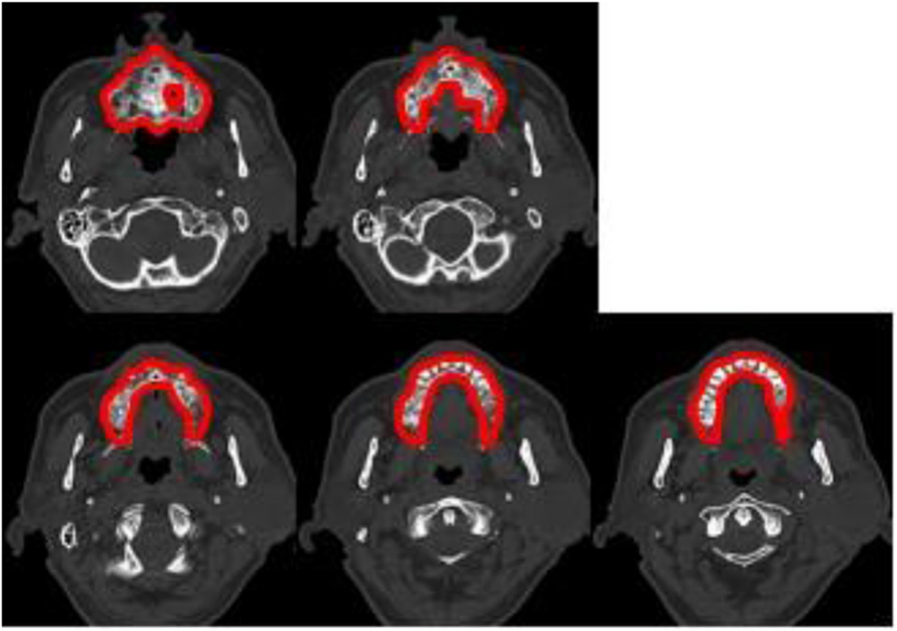
**Representative contouring of the maxilla.** All computed tomography images of the contoured maxilla are shown.

DVH analysis was performed to identify the dose-volume effects on and risk factors for maxillary ORN. The maxillary DVH included the alveolar process and palatine process of the maxilla. However, the pterygoid process and maxillary sinus were not included. Additionally, the root portion of each tooth embedded in the maxilla was included, but its crown was not (Figure 2). Cumulative DVH of the maxilla was calculated during treatment planning. Based on the DVH data obtained from HIPLAN software, the maxilla volumes receiving more than 10–50 GyE in 10-GyE increments were expressed as DVH parameters V10–50 (ml). The average volume of the maxilla was 27.0 ml (range, 12.5–49.8 ml).

### Statistical analysis

The follow-up time was calculated from the first date of irradiation. On univariate analysis of different subgroups, cumulative incidence of maxillary ORN was evaluated using the Kaplan–Meier method and compared by the log-rank test. Furthermore, factors with statistical significance on univariate analysis were applied to multivariate analysis using Cox’s proportional hazard model. To compare the irradiated maxilla volumes, the Mann-Whitney U test was used. A *p* value of <0.05 was considered statistically significant. All statistical analysis was performed using the SPSS software version 11 (SPSS Inc., Chicago, IL).

## Results

### Incidence of maxillary ORN

The median follow-up time was 79 months (range, 24–167 months). Of the 63 enrolled patients, maxillary ORN developed in 26 (41.3%). The median interval between treatment initiation and maximum ORN observation was 23 months (range, 6–107 months). Grade 1 ORN was observed in 14 patients; grade 2, in 9, and grade 3, in 3. None of the patients experienced grade 4 ORN. The 3 patients with grade 3 ORN underwent sequestrectomy for pain control. Subsequently, they also received implantation of a denture and palatal prosthesis.

### Risk factors for maxillary ORN

Univariate analysis was performed for clinical and DVH parameters to analyze their effects on the development of grade ≥1 ORN (Table 1). Patients were divided into 2 groups by median values for analysis of both age and DVH parameters. On univariate analysis, large V10 to V50 values, chemotherapy, and the presence of teeth within the PTV were found to correlate with ORN development. However, factors such as age, sex, smoking, and alcohol consumption had no influence on ORN occurrence.

**Table 1:** Univariate analysis of risk factors of osteoradionecrosis

**Factors**	**Subgroup**	**n**	**p value**	**5-y ORN rate (%)**
V10	<14.5 ml	31		26.1
	≥14.5 ml	32	0.015	53
V20	<11.9 ml	31		20
	≥11.9 ml	32	0.001	58.5
V30	<8.1 ml	31		13.7
	≥8.1 ml	32	0.001	64.4
V40	<4.6 ml	31		12.1
	≥4.6 ml	32	0.0002	67.7
V50	<3.0 ml	31		11.7
	≥3.0 ml	32	0.0002	69.5
Age (years)	<59	31		33.5
	≥59	32	0.23	45.5
Sex	Male	31		40.4
	Female	32	0.5	38.2
Teeth in the PTV	None	34		5.9
	Some	29	0.0001	81
Smoking	Yes	22		41.6
	No	41	0.66	38.6
Alcohol	Yes	31		48.6
	No	32	0.11	30.3
Chemotherapy	Yes	23		54.0
	No	40	0.04	30.5

A large V10 to V50 and the presence of teeth within the PTV were found to correlate with ORN development.

Multivariate analysis was performed with the factors that reached statistical significance on univariate analysis: V10 to V50 values, chemotherapy, and the presence of teeth within the PTV. All data on DVH parameters were evaluated as continuous variables, as opposed to discrete cutoff points used in univariate analysis. Multivariate analysis revealed that V50 (p = 0.0009; hazard ratio [HR] = 1.15; 95% confidence interval [CI] = 1.06–1.25) and the presence of teeth within the PTV (p = 0.0001; HR = 11.3; 95% CI = 3.25–39.35) were significant independent risk factors for ORN (Table 2).

**Table 2:** Multivariate analysis of risk factors of osteoradionecrosis

	**Hazard ratio**	**95% confidence interval**	**p value**
V50	1.15	1.06–1.25	0.0009
Teeth in the PTV	11.3	3.25–39.35	0.0001

V50 and the presence of teeth within the PTV were significant independent risk factors for ORN.

### Correlation between DVH parameters and ORN

**Figure 3:**
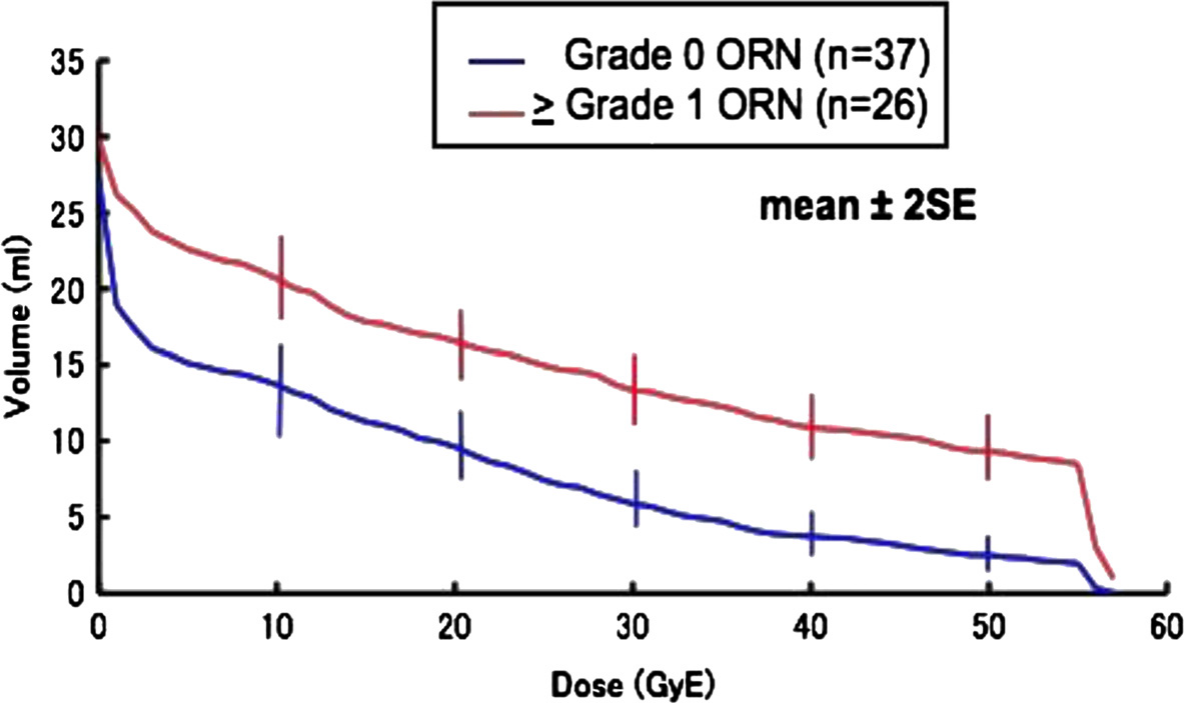
**The average maxillary volumes receiving 10 to 50 GyE according to the occurrence of osteoradionecrosis.** All DVH parameters were significantly higher in patients with ORN than in those without.

Figure 3 shows the mean percentages of DVH parameters from V10 to V50 in patients with and without ORN. All DVH parameters were significantly higher in patients with ORN than in those without ORN (Table 3).

**Table 3:** Correlation between dose-volume histogram parameters and osteoradionecrosis

**Parameters (mean, ml)**	**ORN**	**No ORN**	**p value**
V10	20.68	13.71	0.0054
V20	16.55	9.64	0.0004
V30	13.34	5.86	<0.0001
V40	10.90	3.73	<0.0001
V50	9.32	2.48	<0.0001

All DVH parameters were significantly higher in patients with ORN than in those without ORN.

## Discussion

ORN of the maxilla is a serious adverse event in patients receiving C-ion RT for head and neck tumors. Therefore, it is clinically important to define the risk factors for ORN. In the present study, we demonstrated that ORN development after C-ion RT correlated with dosimetric parameters. In addition, our results confirmed that V50 and the presence of teeth within the PTV were independent risk factors for ORN.

Several previous studies have reported the effects of dosimetric parameters on ORN prediction [4,6-8]. Tsai *et al*. [6] reviewed 402 oropharyngeal cancer patients treated with definitive photon RT using three-dimensional conformal radiotherapy or intensity modulated radiotherapy for ORN and revealed that V40, V50, and V60 were risk factors for ORN on univariate analysis, whereas V50 and V60 were also risk factors on multivariate analysis. On the basis of such findings, they concluded that minimizing the percentage of bone volume exposed to 50 Gy could reduce ORN risk. In our study, although a 16-fraction protocol was used for C-ion RT, V50 remained a statistically significant independent risk factor for ORN. If the predetermined RBE value was correct, and the linear-quadratic model could be applied to C-ion RT, 50 GyE in 16 fractions would be equivalent to 61.1 GyE at a standard fractionation of 2 GyE per fraction with a 3-Gy α/β value.

Chang *et al.*[7] reported that a radiation dose of more than 70 Gy was associated with an increased risk for ORN. Ben-David *et al.*[4] and Studer *et al*. [8] suggested that ORN might be related to mandibular volumes receiving high doses as the situation could exacerbate bone exposure caused by severe acute mucositis. Therefore, a reduction of the bone volume exposed to a high radiation dose may prevent ORN. When comparing the DVH parameters between patients with ORN and those without, a significant difference was observed even at lower radiation doses. However, the effects of low radiation dose on the occurrence of ORN remain unclear. In the above-mentioned study by Tsai *et al.*[6], V10 to V30 were not risk factors for ORN on univariate analysis. In our study, however, all DVH parameters including V10 to V30 were risk factors for ORN on univariate analysis, although only V50 was confirmed as a risk factor by multivariate analysis. The difference between C-ion RT and photon RT data for low doses might result from dose distribution features of these modalities. In three-dimensional conformal radiotherapy and intensity modulated radiotherapy, low-dose regions are generally enlarged because multi-portals are used to ensure conformable dose distribution to the target. On the other hand, C-ion RT can deliver conformable dose distribution to the target without expanding the low-dose regions [13]. Therefore, the maxilla volumes irradiated at low doses in C-ion RT might actually correlate with those receiving higher doses.

In our study, the presence of teeth within the PTV was indicated as an independent risk factor for ORN after C-ion RT. The correlation between the presence of teeth within the PTV and ORN in photon RT has also been reported. Morrish *et al.*[14] reported that ORN developed in 19 of 78 (24.4%) patients with in-field teeth, whereas only 3 of 22 (13.6%) patients without in-field teeth experienced the condition. Additionally, Chang *et al*. [7] reported that the rate of ORN for patients with in-field teeth was 32/239 (13.4%), while it was only 5/174 (2.9%) for those without in-field teeth. ORN has been suggested to develop as a result of gingiva regression by irradiation, leading to an environment that is susceptible to bone infection [15]. In fact, ORN development was found around the tooth in several patients after C-ion RT. Thus, oral care after treatment is very important, especially in cases when teeth are included in the PTV.

Most available reports on ORN after photon RT to head and neck tumors have focused on the mandible. In the present study, we instead investigated ORN of the maxilla because tumors treated with C-ion RT were primarily located in the sinonasal cavity. However, our results on ORN of the maxilla were similar to previous findings on the mandible. Although the mandible is also irradiated in C-ion RT for sinonasal tumors, the radiation dose delivered to the mandible could be reduced owing to its superior physical spatial distribution.

In our study, ORN was observed on the basis of MRI findings in 26 of 63 patients (41.3%) during a median follow-up time of 79 months. Owing to the sensitivity of MRI, 14 of the 26 patients were diagnosed with grade 1 ORN and exhibited no clinical symptoms. Grade 3 ORN was observed in only 3 patients.

Since the present study aimed to investigate the effects of irradiation on ORN development in patients treated with C-ion RT, patients with tumor invasion to the maxilla before C-ion RT were excluded, as it may be challenging to distinguish between osteomyelitis caused by tumor invasion and ORN.

## Conclusion

Our findings from the present study are useful for predicting the risk of ORN and therefore preventing the development of such a condition after C-ion RT for head and neck tumors. We found that ORN development after C-ion RT was correlated with dosimetric parameters. In addition, V50 and the presence of teeth within the PTV were risk factors for the development of ORN after C-ion RT. Such findings confirmed the dose-volume effects on ORN development.

## Abbreviations

C-ion RT: Carbon ion radiotherapy; ORN: Osteoradionecrosis; DVH: Dose-volume histogram; GyE: Gray equivalent; PTV: Planning target volume; CT: Computed tomography; RBE: Relative biological effectiveness; MRI: Magnetic resonance imaging; V10-50: Maxilla volumes receiving more than 10–50 GyE; n: Number.

## Competing interests

The authors declare that they have no competing interests.

## Authors’ contributions

MK and AH conceived the study. GS, HI, and RT participated in data collection. GS performed statistical analysis and drafted the manuscript. MK helped drafting the manuscript. TK and YO critically reviewed the manuscript. All authors read and approved the final manuscript.
